# Resilience, coping, and distress among healthcare service personnel during the COVID-19 pandemic

**DOI:** 10.1186/s12888-021-03506-6

**Published:** 2021-10-06

**Authors:** Timothy R. Elliott, Paul B. Perrin, Anne-Stuart Bell, Mark B. Powers, Ann Marie Warren

**Affiliations:** 1 Department of Educational Psychology, Texas A&M University, College Station, TX 77843-4225 USA; 2 Department of Psychology, Virginia Commonwealth University, Richmond, Virginia USA; 3 Baylor University Medical Center, Dallas, TX USA; 4 Baylor Scott & White Research Institute, Baylor University Medical Center, Dallas, TX USA

**Keywords:** COVID-19, Healthcare workers, Resilience, Coping, Depression, Anxiety

## Abstract

**Background:**

The COVID-19 pandemic has a detrimental effect on the health and well-being of health care workers (HCWs). The extent to which HCWs may differ in their experience of depression and anxiety is unclear, and longitudinal studies are lacking. The present study examined theorized differences in distress between resilient and non-resilient HCWs over time, as reported in a national online survey. We also examined possible differences in distress as a function of sex and doctoral-level status.

**Methods:**

A national sample responded to an online survey data that included the study measures. Of the HCWs who responded, 666 had useable data at the two time points. A longitudinal structural equation model tested an a priori model that specified the relationship of a resilient personality prototype to self-reported resilience, coping, depression and anxiety at both measurement occasions. Additional invariance models examined possible differences by sex and doctoral-level status.

**Results:**

The final model explained 46.4% of the variance in psychological distress at Time 1 and 69.1% at Time 2. A non-resilient personality prototype predicted greater depression and anxiety. A resilient personality prototype was predictive of and operated through self-reported resilience and less disengaged coping to effect lower distress. No effects were found for active coping, however. The final model was generally invariant by sex and HCWs status. Additional analyses revealed that non-doctoral level HCWs had significantly higher depression and anxiety than doctoral-level HCWs on both occasions.

**Conclusions:**

HCWs differ in their susceptibility to distress imposed by COVID-19. Those who are particularly vulnerable may have characteristics that contribute to a lower sense of confidence and efficacy in stressful situations, and more likely to rely on ineffective, disengaged coping behaviors that can exacerbate stress levels. Individual interventions and institutional policies may be implemented to support HCWs at risk.

## Background

The devastating effects of the novel coronavirus disease 2019 (COVID-19) on personal health and well-being are well established. As a “broad-based stressor,” the pandemic exerts a deleterious impact at “… the individual, familial, community, and population level” [1]. Consequently, the COVID-19 pandemic has been associated with increased rates of depression and anxiety throughout the United States, based on the 2018 and 2020 National Health Interview Surveys [2], and available data from sources worldwide reveal similar rates of depression and anxiety as well as other behavioral disruptions in everyday routines [3].

COVID-19 imposes a disproportionate risk of infection and distress on healthcare service personnel [4, 5]. Healthcare workers (HCWs) are vulnerable to infectious epidemic and pandemic outbreaks, generally [6], and those who work in overcrowded conditions, first-responders, and those who have prolonged, direct face-to-face contact with patients are at greater risk to experience emotional distress [7]. Several recent reviews of the emerging literature of mental health issues among HCWs during the COVID-19 pandemic observe a wide variation in the reported rates of depression (from 5 to 51%) and anxiety (14.5 to 44.6%) across studies, most of which are cross-sectional [6, 8–10]. Despite this variation, these reviews concur that nurses and other HCWs with direct, routine patient contact are more likely than physicians and other doctoral-level providers to report clinically significant levels of depression and anxiety, with women more at risk than men. These patterns are confounded by the fact that, according to the U.S. Bureau of Labor Statistics, 87% of nurses and approximately half of physicians are women [11], and women constitute over 74% of all hospital employees [12]. The stressors imposed by the multiple role conflicts and inequities these women experience in managing their professional and personal responsibilities may contribute to their level of risk [13].

There is also evidence in the literature that certain psychological factors might distinguish those who appear particularly vulnerable to severe distress under these circumstances, from those who are not. In their review of the relevant literature, Preti et al. observed that maladaptive coping behaviors and neuroticism were unique risk factors for distress among HCWs during epidemics, and indicators of personal resilience seem to serve a protective role [6]. Cross-sectional studies of community samples during the current pandemic find that passive [14] and negative coping behaviors (i.e., wishful thinking, denial, avoidance, substance use) are associated with higher depression, anxiety and distress [15]. More specifically, avoidant coping [16] and problematic alcohol use [17] are associated with higher distress, depression and anxiety among HCWs. Disengaged, passive coping strategies serve to manage or avoid the negative emotions elicited by stress [18], while active, problem-focused coping has been associated with less distress among community residents [14]. Although resilience is often observed among individuals under duress [19], only one study of self-reported resilience among HCWs found that it was associated with less distress among inexperienced staff and not the more experienced [20]. Studies with community samples have found associations between self-reported resilience and distress in expected directions [14, 21].

Theoretically and clinically, it is important to examine the potentially beneficial role of resilience among HCWs. According to Block [22, 23], ego control and ego resiliency develop from healthy attachments during infancy and, if nurtured throughout childhood, they imbue an individual with the capacities and resourcefulness required to effectively adapt to life transitions, changes and stress. Longitudinal research spanning the past few decades has documented that individuals who have personality traits that characterize a resilient prototype demonstrate prosocial, proactive and self-regulatory behavior, greater cognitive flexibility, increased engagement in goal-directed and emotionally rewarding activities, and more optimal physical and emotional health as they age into adulthood [24–27]. Recent work guided by this model finds similar patterns among clinical populations. One cross-sectional investigation found individuals with acquired disabilities who were not resilient had a more negative orientation toward solving routine, stressful problems and displayed more dysfunctional problem-solving abilities than those who were resilient [28]. Longitudinal studies of warzone veterans using contextual statistical models reveal that resilience exerts beneficial effects through psychological flexibility to facilitate adjustment [29, 30]. Veterans who were characterized as non-resilient, however, consistently relied on avoidant coping strategies that were, in turn, significantly predictive of problems with depression, post-traumatic stress, functional impairment, and a lower quality of life. Alternatively, resilient veterans are more likely to report adaptive health and sleep behaviors, more stress management techniques and a greater tolerance for emotional distress, and they see themselves as resilient under stress compared to those with a non-resilient personality prototype [31]. Longitudinal studies that examine mediators are particularly important because they isolate specific mechanisms through which resilience operates that can be then addressed in interventions to help others learn how to become resilient in routine and stressful conditions.

We conducted the present study to understand the possible benefits of a resilient prototype among HCWs during the current pandemic. Relying on the Block model of personality development, we examined longitudinal data from a national online survey that included an instrument from which we could derive resilient and non-resilient personality prototypes and tested an a priori model reflecting our theoretical assumptions. Extrapolating from prior research, we hypothesized that HCWs with a resilient personality prototype would report more active coping strategies and a greater sense of their own personal resilience than those characterized as non-resilient. We also expected HCWs with a non-resilient prototype would report more disengaged, avoidant coping behaviors. Through these mechanisms we then expected non-resilient HCWs to have more symptoms of depression and anxiety than resilient HCWs. We also examined the possible differences in these relationships as a function of gender, and between doctoral-level and non-doctoral level HCWs.

## Methodology

### Procedure

The study was approved by the Baylor Scott & White Institutional Review Board (IRB#020–235). All methods were carried out in accordance with the relevant guidelines and regulations. Participants were recruited using Qualtrics Panels, a survey sampling and administration company. Qualtrics eliminates responses from individuals on a variety of factors including those with incomplete surveys, straight lined responses or inconsistent responses, and those who responses occurred under half of the median time. Individuals who were 18 years of age and older and could complete the survey in English were the only inclusion criteria. The survey was distributed in two waves to a United States representative sample. The first occurred following IRB approval in June 22nd – July 5th, 2020. The second assessment was conducted 3 months later (from September 22nd to October 19th, 2020). Participants gave informed consent to proceed to the survey.

### Participants

Following recommended practices for using online surveys, we carefully examined the data for missingness and attrition. Initially, 1419 health care providers participated in the study at Time 1, although 748 (52.7%) did not participate at Time 2. Because the focus of this study was on longitudinal effects and imputing these participants’ missing Time 2 scores would generate tremendous predictive error, their data were removed from further analyses. An additional five participants who took the survey at both time points had incomplete on the measures used in this study, and their data were removed as well, yielding a final sample size of *n* = 666. The remaining participants had extremely low levels of missingness (0.3% across the sample) and had item-level data for all scales. The final sample of 666 participants, as shown in Table 1, was primarily comprised of female and White HCWs, with 82.1% of the sample endorsing a non-doctoral education level.

**Table 1:** Summary of Participant Characteristics

Characteristics		
Age, *M*, *SD*	51.49	12.62
Years as Health Care Worker, *M*, *SD*	22.74	13.14
Had Tested Positive for COVID-19, *N, %*		
Time 1	13	5.5
Time 2	20	6.0
Had Been around COVID-19 Patients, *N, %*		
Time 1	258	38.7
Time 2	270	48.2
Sex, *N*, %		
Male	195	29.3
Female	471	70.7
Race/Ethnicity, *N*, %		
White	549	82.4
Asian	60	9.0
Hispanic	26	3.9
Black	45	6.8
Other	4	.6
Native Hawaiian or Pacific Islander	2	.3
Yearly Household Income, *N*, %		
$10,000 to $19,999	12	1.8
$20,000 to $29,999	13	2.0
$30,000 to $44,999	47	7.1
$45,000 to $59,999	57	8.6
$60,000 to $74,999	66	9.9
$75,000 to $99,999	122	18.3
$100,000 to $149,999	141	21.2
$150,000 or More	170	25.5
Prefer not to answer	38	5.7
Education Level, *N*, %		
Non-Doctoral	547	82.1
Doctoral	119	17.9

*Note*. Education level was reported via three options with the first classified as doctoral level and the other two specified as non-doctoral level: “Physician (GP, surgeon, dermatologist, dentist, psychologist, pediatrician, optometrist, orthodontist, etc.),” “Nurse (LPN, RN, NP, Asst, etc.)” and “Other healthcare professional (Laboratory, housekeeping, medical records, nutritionist, therapist, pharmacy, social worker, hospice, etc.,).” Not all categories add up to 666 participants because of skipped responses

### Measures

To test our theoretical model, we used measures of common personality traits, coping, self-reported resilience, depression, and anxiety that were embedded in the survey.

#### Personality prototypes

The Ten-Item Personality Inventory (TIPI) is a well validated brief measure of the “Big Five” personality traits of Extraversion (E), Agreeableness (A), Conscientiousness (C), Openness to Experience (O), and Emotional Stability (ES; the inverse of neuroticism) [32]. The instrument was designed to efficiently assess these traits under time constraints and in survey research. Cluster analysis of the separate trait scores is used to derive the resilient and non-resilient prototypes [30]. Two-item correlation coefficients for the five subscales in the current study were: Extraversion (*r* = .51), Agreeableness (*r* = .32), Conscientiousness (*r* = .34), Emotional Stability (*r* = .53), and Openness to Experience (*r* = .22).

#### Coping

The 28-item Brief COPE (B-COPE) assessed coping styles at the two time points [18]. Items are rated on a four-point Likert-type scale, ranging from 0 (“I haven’t been doing this at all”) to 3 (“I’ve been doing this a lot”). In line with prior factor analyses [33], Active and Disengaged subscales were calculated. Higher scores on each subscale reflect a greater proclivity for that coping style. Cronbach’s αs in the current sample across the two measurement occasions for the Active Coping subscale were .87 and .88, respectively, and for the Disengaged Coping subscale were both .84.

#### Self-reported resilience

The Connor-Davidson Resilience Scale 10 (CDRISC [34]) is a self-report questionnaire containing ten items rated on Likert-type scale (ranging from “*not true at all*” to “*true nearly all the time*”). The total score ranges from 0 to 40; higher scores reflect a greater sense of personal resilience under stress. The CDRICS has demonstrated excellent psychometric properties and it appears to assess the stable, confident element of resilience as described in the Block model [35]. However, longitudinal research suggests that CDRISC scores may fluctuate with changes in emotional adjustment and social support [36], and the predictive value of the total score can be obviated in contextual models that take into account variance attributable to stable personality traits and other self-reported characteristics [29]. Cronbach’s αs in the current sample at the two measurement occasions were .92 and .94, respectively.

#### Depression and anxiety

The Patient Health Questionnaire-8 (PHQ-8 [37]) has eight items that assess symptoms associated with a major depression syndrome. Respondents indicate the degree to which each item was a problem over the past two weeks, using on a four-point Likert-type scale (ranging from “*not at all*” to “*nearly every day*”). Higher scores represent a greater number and severity of symptoms. A score of 10 or more indicates a moderate level of depression, and a score ≥ 15 indicates severe depression [38]. Cronbach’s αs in the current sample across the two measurement occasions were both .90.

Anxiety was assessed with the Generalized Anxiety Disorder scale (GAD-7 [39]). The GAD-7 has seven items that correspond to symptoms associated with a generalized anxiety disorder, and these items are rated on a four-point Likert-type scale (ranging from "*not at all*" to "*nearly every day*"). A higher total score indicates greater symptomology; moderate and severe levels of anxiety are determined by the same cutoff scores used for the PHQ-8 [38]. Cronbach’s αs in the current sample across the two measurement occasions were both .93.

### Data analysis

For the 666 retained participants, the expectation maximization algorithm was used to impute missing data within a scale at the item level using other available items within both time points. Expectation maximization is a modern imputation technique using a maximum likelihood approach, iteratively employing all available data to calculate a particular value for a missing data point. It therefore considers the overall patterns of values in the full sample, as well as the patterns for an individual participant, generating a predicted value tailored to a participant from multivariate data patterns.

In order to quantify HCWs anxiety and depression levels over time, percentages were calculated for each time point based on the number of participants who met or surpassed a threshold score of 10, reflecting moderate or severe symptom levels. A correlation matrix was calculated to examine the bivariate relationships among all primary study constructs, with means and standard deviations. To derive the resilient prototype variable, a *k*-means cluster analysis was conducted following Meyers, Gamst, and Guarino’s [40] procedure in SPSS version 27.0. A cluster analysis uses classifying variables -- here the five TIPI subscale scores at Time 1 -- to sort participants into groups. The TIPI subscale scores were first transformed into *z*-scores before being entered into the cluster analysis. Univariate analyses of variance (ANOVAs) examined whether the clusters differed on the five TIPI subscales. Consistent with the literature on the resilience prototype [30], two clusters were specified in the analysis to distinguish resilient and non-resilient groups.

**Fig. 1:**
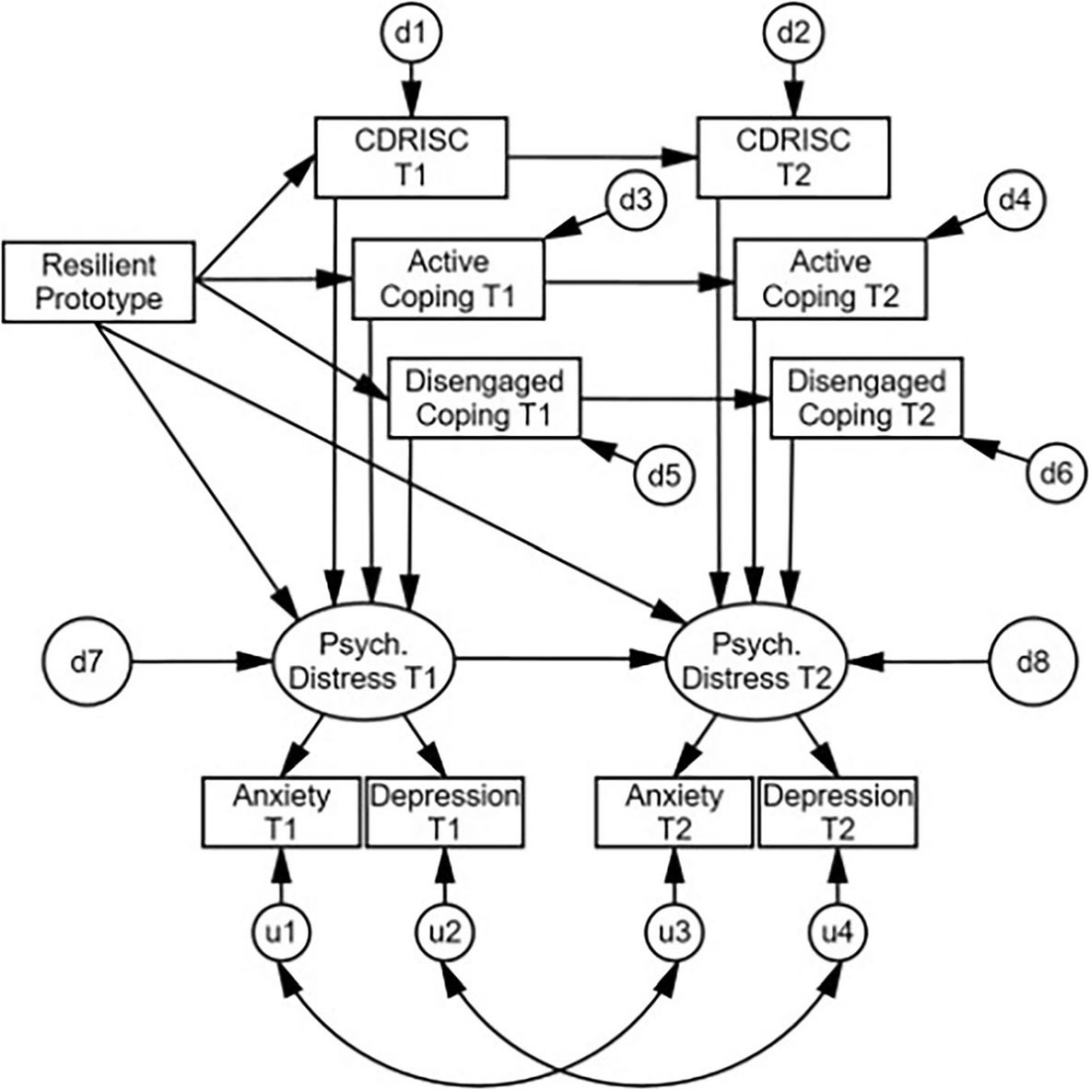
A priori Theoretical Model of Personality Prototypes, Mediators and Distress

A longitudinal structural equation model (SEM) was then created using AMOS 23.0 based on the theory articulated above linking the resilient prototype to mental health at Time 1 and 2 via active coping, disengaged coping and self-reported resilience at the two time points. A visual representation of the initial theoretical model appears in Fig. 1, as it would be analyzed in an SEM. All three sets of longitudinal mediators were specified to be simultaneous mediators, though within each mediator, time was specified sequentially (e.g., resilience at Time 1 leading to resilience at Time 2). The two latent variables in the SEM were mental health at Time 1 and Time 2, each created from the manifest variables of anxiety and depression at each respective time point (with the uniqueness terms for anxiety and depression allowed to correlate over time and designated with a “u”). All other variables were specified to be manifest, with appropriate disturbance terms drawn for all endogenous variables (designated with a “d”). The following criteria were used to assess goodness of fit [41]: goodness of fit index (GFI), adjusted goodness of fit index (AGFI), normed fit index (NFI), incremental fit index (IFI), and Tucker-Lewis index (TLI) > .90 [42, 43]; comparative fit index (CFI) > .95 [43]; and a root mean squared error of approximation (RMSEA) of < .10 [40]. The analytic procedure started with the full model as shown in Fig. 1. Following the trimming procedure recommended by Meyers, Gamst, and Guarino [40], all non-significant paths and the variables driving that lack of significance if they did not connect to the rest of the model were eliminated. Once a final model was retained, all possible indirect effects and bias-corrected significance levels were calculated using 2000 bootstrap samples. The retained SEM was run with two overall tests of invariance for sex (male vs. female) and health care provider status (doctoral level vs. non-doctoral level) to determine if the model held equally well across these groups.

## Results

### Preliminary analyses and descriptive statistics

We conducted preliminary analyses to detect any differences between the sample retained for this study (*n* = 666) from those who were excluded (*n* = 753). A series of analyses of variance (ANOVAs) were conducted on the primary variables at Time 1. Only the raw (rather than imputed when missing) values were used in these analyses. The two groups of participants did not differ significantly on anxiety, active coping, disengaged coping, resilience, extraversion, agreeableness, or emotional stability (all *p*s ≥ .05). However, those who were retained for this study reported lower depression (*p* = .048, η^2^ = .003), higher conscientiousness (*p* = .002, η^2^ = .007), and lower openness (*p* = .002, η^2^ = .007).

**Fig. 2:**
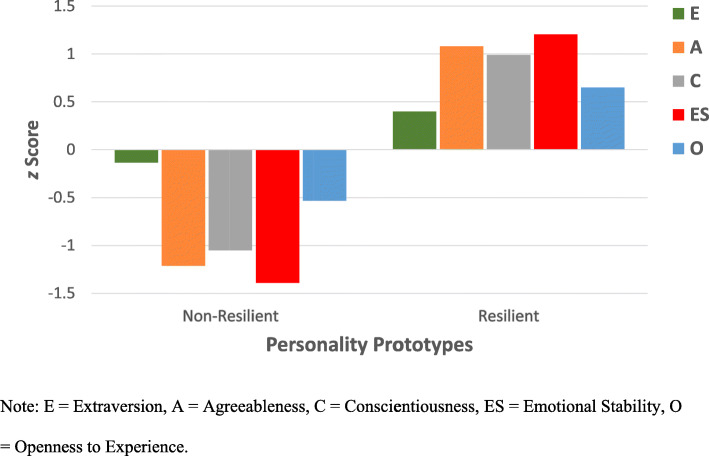
Resilient (*n* = 404) and Non-Resilient Personality (*n* = 262) Prototypes among HCWs Based on TIPI Cluster Center z Scores. Note: E = Extraversion, A = Agreeableness, C = Conscientiousness, ES = Emotional Stability, O = Openness to Experience

To identity the personality prototypes, participants’ TIPI subscale scores at Time 1 were converted to *z*-scores and analyzed using a k-means cluster analysis with a 2-group cluster specification. Convergence was reached in 8 iterations. Univariate ANOVAs indicated that the cluster groups differed significantly on all five TIPI subscale scores (all *p*s < .001). The final cluster centers together with the number of participants in each cluster are shown in Table 2. Participants in Cluster 1 had the lowest score on emotional stability, a defining characteristic of individuals with non-resilient personality prototype. They also reported lower than average scores on the other four traits. As reflected by the *z* scores of the cluster center values depicted in Fig. 2, participants in the resilient personality prototype exhibited the expected elevations in emotional stability, agreeableness, conscientiousness, extraversion, and openness to experience. This pattern is consistent with personality clusters observed among clinical samples that used other measures of these five personality traits [28, 30]. In subsequent analyses, a resilient personality prototype was coded as 1 and the non-resilient prototype as 0.

**Table 2:** Final Cluster Centers and Cluster Sample Sizes

Classifying Variable	Cluster 1: Non-Resilient (*n* = 262)	Cluster 2: Resilient (*n* = 404)
Extraversion	−.18	.12
Agreeableness	−.78	.50
Conscientiousness	−.69	.45
Emotional Stability	−.88	.57
Openness	−.40	.26

On the PHQ-8, 11.4% of participants at both Time 1 and Time 2 had a total score of 10 or higher, reflecting moderate or severe depressive symptoms. Conversely, on the GAD-7, 10.1% of participants at Time 1 and 11.0% at Time 2 had a total score of 10 or higher, similarly reflecting moderate or severe anxiety symptoms. A correlation matrix showing the bivariate relationships among all primary study variables appears in Table 3. The variables were generally associated with each other as would be expected, with the exception of active coping not being associated with the resilient prototype at either time period. Active coping was associated with self-reported resilience at Time 1 and Time 2. Further, active coping was positively associated with anxiety and depression at both time points, as well as with disengaged coping. Repeated-measures ANOVAs suggested that anxiety and depression scores remained constant over time, although there were slight drops in resilience (*p* = .038), active coping (*p* < .001), and disengaged coping (*p* = .029).

**Table 3:** Correlation Matrix of Variables Included in the Model

Variable	1	2	3	4	5	6	7	8	9	10	11
1. Resilient vs. Non-Resilient Prototype											
2. CDRISC T1	.510*										
3. Active Coping T1	.033	.133*									
4. Disengaged Coping T1	−.325*	−.257*	.491*								
5. Anxiety T1	−.370*	−.353*	.200*	.536*							
6. Depression T1	−.354*	−.338*	.186*	.578*	.747*						
7. CDRISC T2	.432*	.658*	.058	−.269*	−.297*	−.302*					
8. Active Coping T2	.023	.073	.531*	.206*	.133*	.117*	.167*				
9. Disengaged Coping T2	−.287*	−.259*	.254*	.554*	.431*	.502*	−.241*	.516*			
10. Anxiety T2	−.371*	−.330*	.170*	.449*	.718*	.645*	−.328*	.240*	.567*		
11. Depression T2	−.321*	−.316*	.142*	.421*	.635*	.710*	−.327*	.134*	.538*	.791*	
Variable *M*	–	29.71	35.68	19.36	3.45	3.75	29.20	34.48	18.90	3.22	3.52
Variable *SD*	–	7.20	9.33	5.91	4.39	4.67	8.01	9.24	5.74	4.46	4.62

*Note*. * = Correlation is significant at the .01 level (2-tailed). T1 = Time 1; T2 = Time 2. CDRISC = Connor-Davidson Resilience Scale (10 item); Resilient personality prototype coded as 1, non-resilient personality prototype coded as 0

### Longitudinal structural equation model (SEM)

In the initial SEM (Fig. 1; χ^2^[34] = 763.75, *p* < .001), the majority of paths were statistically significant, although there were a number of non-significant paths that called for use of Meyers, Gamst, and Guarino’s [40] trimming procedure: between the resilient prototype and active coping T1 (β = .03, *p* = .391), active coping T1 and psychological distress T1 (β = −.02, *p* = .532), active coping T2 and psychological distress T2 (β = .01, *p* = .740), and between the resilient prototype and psychological distress T2 (β = −.02, *p* = .505). All other paths in the model were statistically significant (all *p*s < .006). Further, the fit indices of the initial SEM were all in the poor range, likely because of the paths that were not statistically significant: GFI = .86, AGFI = .72, NFI = .82, IFI = .83, TLI = .72, CFI = .82, and RMSEA = .18.

**Fig. 3:**
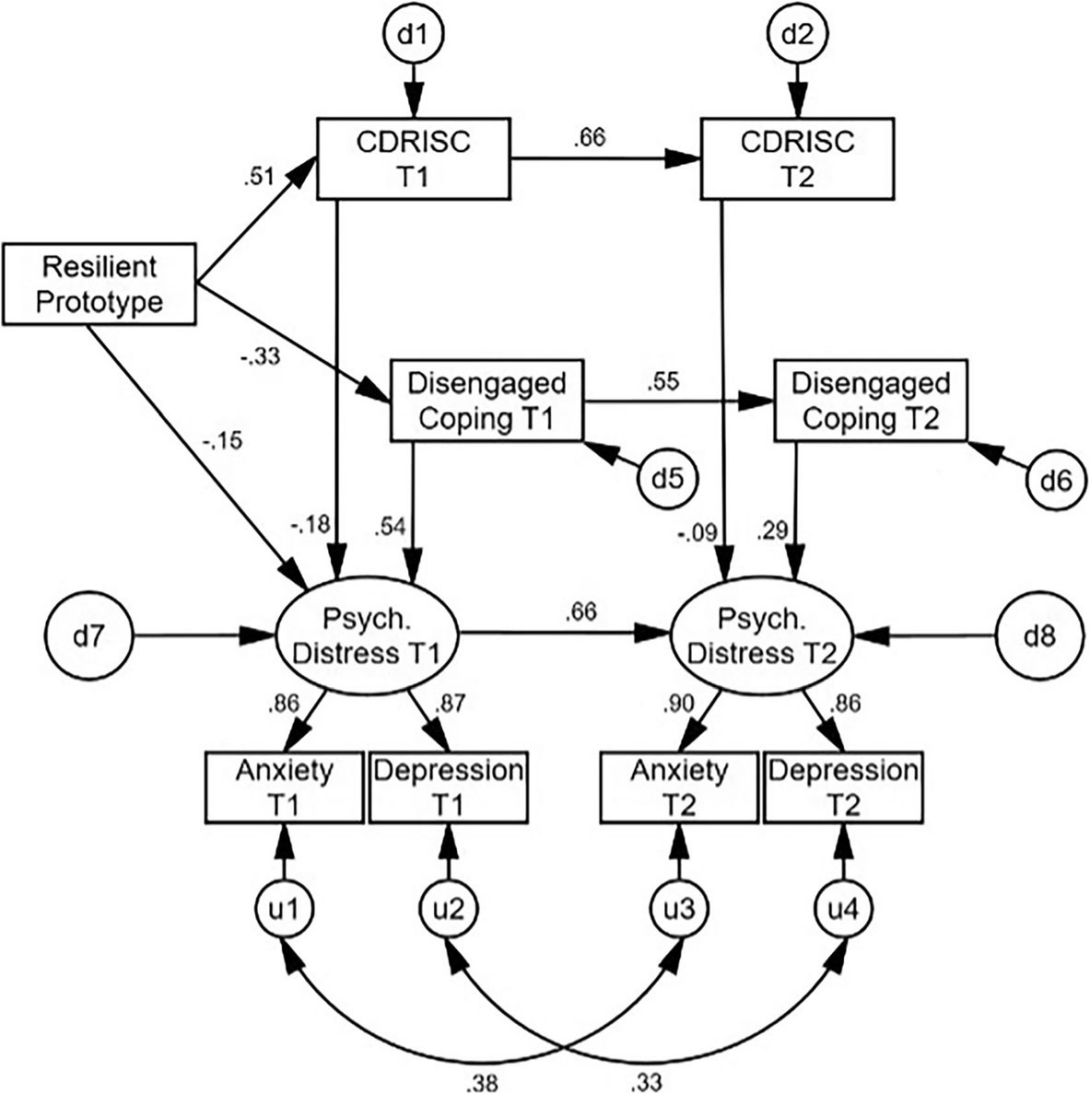
Final Model

Following the trimming procedure, each of these non-significant paths was trimmed (deleted), as well as any variables that no longer connected to the rest of the model (e.g., active coping T1-T2). All paths in the refined SEM (Fig. 3; χ^2^[20] = 133.56, *p* < .001) were statistically significant (all *p*s < .002). The fit indices of the refined model improved dramatically over the initial one, and were either in the good or adequate range [40–43], suggesting overall adequate model fit: GFI = .96, AGFI = .91, NFI = .96, IFI = .97, TLI = .94, CFI = .97, and RMSEA = .09. As a result of this combination of findings, this corrected model was retained as the final model.

In the final model, the resilient personality prototype was positively associated with self-reported resilience at Time 1, which was positively associated with Time 2 resilience. The resilient personality prototype was also inversely associated with disengaged coping at Time 1, which then positively predicted disengaged coping at Time 2. Self-reported resilience at each time point was inversely associated with psychological distress at each respective time point; disengaged coping was positively associated with psychological distress at each respective time point. The standardized and unstandardized path coefficients for the indirect effects in the model are contained in Table 4. All possible indirect effects were statistically significant (β magnitude range = .18–.52, all *p*s < .001), suggesting strong internal structure of the model. In particular, the multiple mediational effects from resilient personality prototype to psychological distress at Time 2 was β = −.36 (*p* < .001), and with the combination of a previously non-significant and now trimmed path from the resilient personality prototype to psychological distress at Time 2, suggested a very significant and full mediation throughout the entire theoretical chain. In sum, a resilient personality prototype was significantly predictive of lower distress at both occasions through its beneficial associations with self-reported resilience, and with less use of disengaged coping, in addition to its direct effect on distress at Time 1. The final model explained 46.4% of the variance in psychological distress at Time 1 and 69.1% of the variance at Time 2.

**Table 4:** Standardized and Unstandardized Path Coefficients and Confidence Intervals of Indirect Effects of Personality Prototypes and Mediating Variables on Distress Over Time

Effect	Unstandardized	Standardized	Unstandardized 90% C.I. (Bootstrap)
Resilient Prototype -- > CDRISC T1 -- > CDRISC T2	5.50	.34	(4.73, 6.33)
Resilient Prototype -- > Disengaged Coping T1 -- > Disengaged Coping T2	−2.12	−.18	(− 2.66, − 1.61)
Resilient Prototype -- > CDRISC T1-T2 and Disengaged Coping T1-T2 -- > Psych. Distress T2	−2.82	−.36	(−3.34, − 2.30)
Resilient Prototype -- > CDRISC T1 and Disengaged Coping T1 -- > Psych. Distress T1	−2.22	−.27	(− 2.70, − 1.77)
CDRISC T1 -- > CDRISC T2 -- > Psych. Distress T2	−.10	−.18	(−.13, −.06)
Disengaged Coping T1 -- > Disengaged Coping T2 -- > Psych. Distress T2	.33	.52	(.28, .39)

*Note*: All indirect effects significant at *p* < .001

An invariance model was conducted as a function of sex (male vs. female). Five sets of comparisons were of interest: measurement weights, structural weights, structural covariances, structural residuals, and measurement residuals. The first three sets of comparisons were non-significant (all *p*s > .066). However, the structural residual comparison did reach statistical significance, χ^2^(19) = 51.65, *p* < .001, suggesting that men and women differed in the magnitude of the disturbance terms in the model. Bonferroni-corrected post-hoc comparisons suggested that only the disturbance terms for disengaged coping at Times 1 and 2 were noninvariant across sex (*z* = − 2.26 and − 3.54, respectively) and larger for men than women at each time point. Also, the measurement residual comparison reached statistical significance, χ^2^(25) = 71.48, *p* < .001, suggesting that men and women differed in the magnitude of the uniqueness terms of the manifest variables in the model. Bonferroni-corrected post-hoc comparisons suggested that only the error term for depression at Time 2 was noninvariant across the two groups (*z* = 2.56) and slightly larger for women than for men. Despite these small caveats, the SEM generally showed overall good invariance across men and women.

A second invariance model was run as a function of HCW status (doctoral level vs. non-doctoral level). The first comparison for measurement weights was non-significant (*p* = .068). However, the structural weight, χ^2^(12) = 34.05, *p* = .001, structural covariance, χ^2^(13) = 34.18, *p* = .001, structural residual, χ^2^(19) = 56.47, *p* < .001, and measurement residual, χ^2^(25) = 113.39, *p* < .001, comparisons all were statistically significant. In terms of structural weights, or path coefficients, once Bonferroni-corrected, post-hoc comparisons suggested that there were no significant differences between doctoral and non-doctoral level HCWs, such that the model was invariant in this regard across the two groups. In terms of structural covariances, or the correlations between error terms in this model, Bonferroni-corrected post-hoc comparisons suggested that only the correlation coefficient between anxiety uniqueness terms at Time 1 and 2 was noninvariant across the two groups (*z* = − 3.25). Upon further inspection, the correlation was *r* = .42 for non-doctoral providers, but *r* = −.05 for doctoral providers, suggesting anxiety levels were more consistent over time for non-doctoral level providers relative to doctoral level HCWs. In terms of structural residuals, Bonferroni-corrected post-hoc comparisons suggested that only the disturbance term for disengaged coping at Time 2 was noninvariant across provider status (*z* = − 2.89), and larger for doctoral level than non-doctoral level HCWs. In terms of measurement residuals, Bonferroni-corrected post-hoc comparisons suggested that the error terms for anxiety and depression at Time 1 were noninvariant across the two groups (*z* = − 3.05 and − 3.92, respectively), and both slightly larger for doctoral than non-doctoral level HCWs. As in the first invariance model, despite these small caveats, the SEM generally showed overall good invariance across doctoral and non-doctoral level HCWs, though there may be some small differences in error terms, and anxiety was likely more consistent over time among the non-doctoral level providers.

We then conducted a repeated-measures multivariate analysis of variance (RMANOVA) and follow-up ANOVAs to determine whether there were differences over time in anxiety and depression as a function of sex or HCW doctoral status. The overall omnibus RMANOVA did not reveal a statistically significant main effect for time, *F* (2, 661) = 1.64, *p* = .194, η^2^ = .005, sex, *F* (2, 661) = 2.36, *p* = .096, η^2^ = .007, nor the sex*time interaction, *F* (2, 661) = 1.10, *p* = .333, η^2^ = .003. However, there was a statistically significant main effect for HCW doctoral status, *F* (2, 661) = 3.62, *p* = .027, η^2^ = .011: Doctoral level providers reported lower anxiety at each time point (*M*s [*SD*s] = 2.45 [3.31] and 1.76 [2.91], respectively) than non-doctoral level providers (*M*s [*SD*s] = 3.67 [4.56] and 3.54 [4.67], respectively), *p*s = .006 and < .001, respectively. Similarly, doctoral level providers reported lower depression at each time point (*M*s [*SD*s] = 2.67 [3.67] and 2.34 [3.74], respectively) than non-doctoral level providers (*M*s [*SD*s] = 3.98 [4.83] and 3.77 [4.75], respectively), *p*s = .006 and .002, respectively. There was no significant HCW status*time interaction, *F* (2, 661) = 2.08, *p* = .126, η^2^ = .006, sex*HCW status interaction, *F* (2, 661) = 1.41, *p* = .246, η^2^ = .004, nor a time*sex*HCW status interaction, *F* (2, 661) = .93, *p* = .397, η^2^ = .003.

Finally, we conducted χ^2^ tests to determine whether the percentages of participants by sex or HCW status differed in the resilient vs. non-resilient personality group. Resilient and non-resilient personality prototype groups did not differ statistically in their proportions of doctoral level and non-doctoral level HCWs, χ^2^(1) = .99, *p* = .319, nor in their proportions of men and women, χ^2^(1) = .05, *p* = .822.

## Discussion

Our final model provides partial confirmation of our hypotheses. A resilient personality prototype was associated with higher self-reported resilience and lower disengaged coping at both time points. The direction and nature of these relationships are enlightening. Our model indicates that those with a non-resilient personality prototype consistently relied on disengaged coping strategies that, in turn, were predictive of greater depression and anxiety. In contrast, those with a resilient personality prototype reported greater self-confidence in their ability to manage stress (as measured by the CDRISC [35]). These resilient individuals did not, however, rely on active coping strategies as we expected. Analysis of indirect effects reveals that the resilient personality prototype operated through these two mediating factors - self-reported resilience and disengaged coping - to exert its effects on depression and anxiety. These indirect, mediating effects are critical to understanding the mechanisms through which resilience facilitated adjustment [44] in a manner that can inform meaningful interventions to help others obtain the skills and resources required to be resilient under stress. Our final model indicates that the beneficial influence of a resilient personality prototype operates among men and women in the sample, and for HCWs regardless of their doctoral-level status.

We know from previous research that individuals described as “trait resilient” possess more stress management skills, healthier sleep patterns, and better tolerate distressing emotions than those who are not [31]. Further, individuals who are trait resilient exhibit a quicker cardiovascular recovery to baseline levels following negative emotional stimuli than those who are not resilient [45]. These differences do not appear to be function of any attentional bias: Individuals who are not resilient appear to become preoccupied with negatively-valenced information, but resilient individuals can disengage from it [46] and regulate their affective experience to meet the demands imposed by changes in the environment [47]. Our results imply that resilient individuals likely maintained a sense of efficacy and confidence and were not compelled to rely on avoidant, disengaged coping strategies (e.g., using alcohol, denial, wishful thinking) to manage or distract from negative emotions. Active coping was positively correlated with distress at both time points, but it exerted no predictive value in the final model. Previous research has found inconsistent relationships between active coping and various indicators of adjustment (e.g., quality of life, functional impairment, depression) over time in contextual models that examine other mediating variables (including self-reported resilience; 30). The findings of the present study imply that resilient individuals were not motivated to engage in a measurable coping behavior to manage to any situational demands, contingencies, or emotional reactions elicited by the COVID-19 pandemic. This might reflect the predisposition resilient individuals have to flexibly adapt to circumstances, to regulate their emotions and behavior in routine and stressful situations, and have confidence in their existing repertoire of psychological, interpersonal and social resources [23].

Contrary to our expectations, women did not report significantly higher levels of depression and anxiety at either measurement occasion. We cannot account for the lack of sex differences in distress in light of the extant literature. Additionally, doctoral level HCWs reported less depression and anxiety than non-doctoral level providers. As the final model was generally invariant as a function of doctoral-level status, we suspect the differences in adjustment observed between doctoral and non-doctoral level HCWs reflect the differentials that exist in the structure, supports and functions of these individuals within the healthcare system. Primarily, these exist beyond the realm of personal control and volition.

### Limitations

The current study has a number of important limitations. Although the participants who were included and excluded in the current study were largely similar across most primary study variables, there was likely an oversampling of those with slightly lower depression, higher conscientiousness, and lower openness. Although these differences might be understandable (e.g., participants who completed the survey were highly motivated and able to do so), they limit the generalizability of the findings. We realize that survey research tends to find higher reported rates of depression and anxiety than found in rigorous studies that feature structured, face-to-face interview systems [48]. The rates of depression and anxiety we found using the recommended cut-off scores for the PHQ-8 and the GAD-7 appear to be in a reasonable range, considering the much higher rates reported in previous research.

## Conclusions

HCWs experience a disproportionate risk to their health and well-being during the COVID-19 pandemic. Our findings indicate that some HCWs may be particularly vulnerable under these conditions, lacking confidence in their ability to meet the demands of these stressful circumstances, and relying on avoidant, disengaged coping strategies that ineffectively resolve their negative emotions. These individuals may benefit from interventions that promote emotional self-regulation, develop a greater sense of efficacy and confidence in the ability to cope, and psychological flexibility in responding to changing demands imposed by the pandemic [49]. Further, our study also reveals that non-doctoral level HCWs who probably have routine and ongoing interactions with patients are more likely than doctoral-level providers to experience problems with depression and anxiety. These findings converge with other relevant work [8–10] to underscore the need for health care systems to attend to the issues these providers experience in their routine, repeated and prolonged exposures to COVID-19 (and other epidemic conditions, generally; 6). Future research could examine other mechanisms through which resilience facilitates adjustment that could potentially inform systematic services and programs for HCWs.

## Data Availability

The data that support the findings of this study are available from the Baylor Scott & White Research Institute, but restrictions apply to the availability of these data, which were used under license for the current study, and so are not publicly available. Data are however available from the authors upon reasonable request and with permission of the Baylor Scott & White Research Institute.

## Abbreviations

AGFI: Adjusted Goodness of Fit Index
ANOVA: Univariate Analysis of Variance
B-COPE: Brief COPE
CDRISC: Connor-Davidson Resilience Scale
CFI: Comparative Fit Index
COVID-19: Novel coronavirus disease 2019
GAD-7: Generalized Anxiety Disorder scale
GFI: Goodness of Fit Index
HCWs: Healthcare workers
IFI: Incremental Fit Index
NFI: Normed Fit Index
PHQ-8: Patient Health Questionnaire-8
RMANOVA: Repeated-Measures Multivariate Analysis of Variance
RMSEA: Root Mean Squared Error of Approximation
SEM: Structural Equation Model
TIPI: Ten-Item Personality Inventory
TLI: Tucker-Lewis Index
